# The relationship between mental fatigue and social responsibility among nurses who provided care to patients with coronavirus disease 2019: a cross-sectional study

**DOI:** 10.1186/s12912-023-01429-z

**Published:** 2023-08-10

**Authors:** Soheileddin Salmani, Mohammad Salehpoor Emran, Afsaneh Sadooghiasl, Shima Haghani, Shahzad Pashaeypoor

**Affiliations:** 1 Department of Nursing, Rozbeh Hospital, Tehran University and Medical Sciences (TUMS), Tehran, Iran; 2 Department of Community Health Nursing, School of Nursing and Midwifery, Tehran University of Medical Sciences, Tehran, Iran; 3 Faculty of Medical Sciences, Department of Nursing, Tarbiat Modares University, Tehran, Iran; 4 Nursing Care Research Center, Iran University of Medical Sciences, Tehran, Iran; 5 Department of Community Health and Geriatric Nursing, School of Nursing and Midwifery, Tehran University of Medical Sciences, Tehran, Iran; 6 Community Based Participatory Research Center, Iranian Institute for Reduction of High–Risk Behaviors, Tehran University of Medical Sciences, Tehran, Iran

**Keywords:** Social responsibility, Mental fatigue, Nurses, COVID-19

## Abstract

**Background and Aim:**

Mental fatigue (MF) was a major challenge for nurses during the coronavirus disease 2019 (COVID-19) pandemic. Nurses’ sense of responsibility towards their patients and societies may influence their MF. This study aimed to assess the relationship between MF and social responsibility (SR) among nurses who provided care to patients with COVID-19.

**Methods:**

This cross-sectional descriptive-analytical study was conducted in 2021. Participants were 258 nurses randomly selected from eleven COVID-19 care hospitals in Tehran, Iran. Data were collected using three self-report instruments, namely a demographic questionnaire, the Mental Fatigue Scale, and the Social Responsibility Questionnaire. The SPSS software (v. 16.0) was used to analyze the data at a significance level of less than 0.05.

**Results:**

The mean scores of MF and SR were 31.73 ± 7.35 and 3.45 ± 0.35, respectively. The highest and the lowest scored SR subscales were ethical responsibilities with a mean of 3.67 ± 0.42 and economic responsibilities with a mean of 2.93 ± 0.62. MF had a significant negative correlation with legal responsibilities and a significant positive correlation with economic responsibilities (P < 0.05). The only significant predictor of SR was financial status which significantly predicted 4.3% of the variance of SR (P < 0.05).

**Conclusion:**

More than half of the nurses who provided care to patients with COVID-19 suffered from MF and their mental fatigue had a significant correlation with their legal and economic responsibilities. Healthcare authorities and policymakers need to develop programs to reduce nurses’ MF and improve their satisfaction.

## Background

Mental fatigue (MF) is a psychological status that is associated with mental exhaustion, reduced energy, and altered functioning during a course of cognitive activities [1, 2]. In the medical field, MF is the primary manifestation of fatigue among nurses [3, 4]. Nurses in different areas of the world suffer from high levels of fatigue [5]. A study reported that nurses with twelve-hour shifts had moderate to severe acute fatigue and low to moderate chronic fatigue [6]. Moreover, MF is associated with negative patient outcomes, low care quality, high risk of medication errors, and problems in nurses’ social life [7, 8]. Therefore, considering the important role that nurses play in patient safety [9], it is importance to investigate the factors affecting MF.

Many different factors can affect MF. For example, long work shifts, shift schedule, age group, and work experience are known influential factors on MF [12]. Evidence also shows that MF is among the leading causes of job turnover [13]. Some studies reported that professional commitment and workload have a significant relationship with mental health, job satisfaction, and fatigue among nurses [10, 11]. Moreover, nurses’ sense of responsibility towards patients and safe and quality care delivery can contribute to their MF [12, 13]. A sense of responsibility is one of the nursing professional values characterized by nurses’ professional commitment and their respect for patients’ rights [14].

Social responsibility (SR) is a main aspect of professional ethics in nursing [15, 16]. By definition, SR is engagement in socially responsible behaviors in response to social demands and public needs [17]. SR prevents nurses from negligence in the process of patient care and helps them provide comprehensive patient care, gain patient trust, and enhance patient satisfaction and peace [18, 19]. It also significantly improves the quality of patient care [16]. SR can keep nurses once hired, maintain their confidence and performance, and safeguard them from MF [20].

Epidemics, such as the coronavirus disease 2019 (COVID-19) pandemic, can also contribute to MF among nurses [21]. COVID-19 is a respiratory problem first diagnosed in 2019 in Wuhan, China, and rapidly spread throughout the world [22] so the World Health Organization reported it as the sixth leading health emergency in the world [23]. A major challenge in COVID-19 management was the shortage of competent healthcare providers, particularly nurses, which was associated with a significant increase in their workload and obliged them to suspend most of their personal plans and recreational activities [24, 25]. Moreover, lack of personal protective equipment, lack of an effective approach to COVID-19 management, media pressure, and poor perceived support had negative effects on healthcare providers’ performance and caused their high levels of stress and worry during the COVID-19 pandemic [26–28].

Mental tensions and strains due to the COVID-19 pandemic were associated with severe MF and negatively affected the quality of nursing care services [29]. The results of a study showed a significant correlation between COVID-19 stress, SR, and job burnout, while SR had mediating effects on the correlation between COVID-19 stress and job burnout [30]. Under the pressure of environmental changes, employees may frequently ignore SR and ethics and fail to utilize SR decision-making concepts to deal with their mental fatigue as well as address the expectations of stakeholders and shareholders [31]. Despite the significant effects of the COVID-19 pandemic as environmental changes on nurses’ MF and SR, there are limited data about MF and its relationship with SR among nurses who provide care to patients with COVID-19, and it is necessary to pay attention. The Covid-19 pandemic affected the physical and mental aspects of nurses. On the other hand, SR is one of the essential responsibilities of nurses. Therefore, it is very necessary to investigate in challenging situations such as pandemics whether it can really be related to MF or not. In this regard, targeted interventions can be considered to improve the SR of nurses. Therefore, the present study was conducted to provide further evidence in this area. The study’s aim was to assess the relationship between MF and SR among nurses who provided care to patients with COVID-19.

## Methods and materials

### Study design

This cross-sectional descriptive-analytical study was conducted in August–November 2021, during the fourth and fifth waves of COVID-19 in Iran.

### Study population and setting

**Fig. 1:**
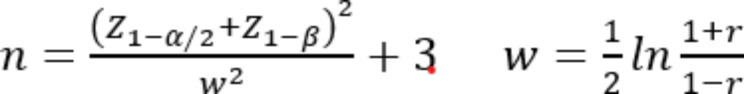
Sample size calculation formula

The study population consisted of all 2000 nurses who provided care to patients with COVID-19 in all eleven COVID-19 care hospitals affiliated with Tehran University of Medical Sciences, Tehran, Iran. Sampling in each hospital was done proportionally according to the population of nurses caring for COVID-19 patients in each hospital. So, a sample of 258 eligible nurses was randomly selected using a table of random numbers and the list of nurses’ names. Considering that the sample size was about 13% of all eligible nurses, therefore about 13% of nurses who met the eligibility criteria were selected from each hospital. Eligibility criteria were a COVID-19 care experience of at least six months, no self-report affliction by chronic diseases or cognitive disorders, and consent for participation in the study. Participants who voluntarily withdrew from the study were excluded. The sample size was calculated with a confidence level of 95%, a power of 80%, and an MF-SR correlation coefficient of at least 0.2 to be considered significant (Fig. 1). The sample size calculation formula indicated that at least 200 participants were needed. Yet, the sample size was increased to 258 to improve the power of the study.

### Instruments and data collection

Data collection instruments were a demographic questionnaire, the Mental Fatigue Scale, and the Social Responsibility Questionnaire. The items of the demographic questionnaire were age, gender, marital status, educational level, financial status, history of affliction by COVID-19, employment status, and work experience.

The Mental Fatigue Scale was developed by Johansson and Starmark in 2010. It has fifteen items on the different aspects of MF, including general fatigue, lack of initiative, MF, mental recovery, concentration difficulties, memory problems, slowness of thinking, sensitivity to stress, increased tendency to become emotional, irritability, sensitivity to light and noise, decreased or increased sleep, and 24-hour symptom variations [32]. Items are scored on a four-point scale as follows: zero: “No problem”; 1: “Problem”; 2: “Relatively serious problem”; and 3: “Serious problem”. The possible total score of the scale is 0–45 with higher scores showing greater MF. In a previous study, seven Ph.D. students of occupational health assessed and confirmed the face and content validity of this scale. Its reliability was also assessed through the test-retest stability and the internal consistency assessment methods which revealed a two-week test-retest Pearson’s correlation coefficient of 0.727 (P = 0.002) and a Cronbach’s alpha of 0.893 [33]. In the present study, ICC was calculated and its value was calculated as 0.94.

The Social Responsibility Questionnaire was developed based on Carroll’s questionnaire with 35 items in four subscales, namely legal responsibilities (seven items), economic responsibilities (seven items), ethical responsibilities (nine items), and discretionary responsibilities (twelve items). Item scoring is done on a five-point scale from 1 (“Completely agree”) to 5 (“Completely disagree”) [34]. A study confirmed the content validity of this questionnaire by seeking the comments of several nursing faculties and confirmed its reliability with a Cronbach’s alpha of 0.86 [18]. Another study also confirmed the construct validity of the questionnaire through the exploratory factor analysis and its reliability with a Cronbach’s alpha of 0.75 [35]. Moreover, a study found that Cronbach’s alpha of the questionnaire was 0.95 [36]. In the current study, ICC was calculated and its value was calculated as 0.87.

In the present study, participants personally completed the printed instruments. This research was approved in the session of the ethics committee of Tehran University of Medical Sciences, with the ethics code IR.TUMS.FNM.REC.1400.027. The researcher went to the department where the nurse was present and when the nurse was ready to complete the questionnaire, the research tool was given to her/him. The purpose and method of research were explained to the nurses, then they were invited to participate in the study, and the written informed consent form was completed by them. Data collection was performed with close adherence to COVID-19 prevention guidelines.

### Data analysis

Data were described through the measures of descriptive statistics (namely frequency, mean, and standard deviation) and were analyzed through the one-way analysis of variance, Pearson’s correlation analysis, and linear regression analysis. Independent variables in the regression analysis were those variables with a significant relationship with MF at a significance level of less than 0.2 [37]. Data analysis was performed using the SPSS software (v. 16.0) and at a significance level of less than 0.05.

## Results

Participants were 258 nurses who provided care to patients with COVID-19 and the response rate was 100%. Most participants were female (69%), had a bachelor’s degree (86%), had average financial status (72.5%), and reported a history of affliction by COVID-19 (54%). Almost half of them were married (50.5%) and had contractual or conditional official employment (48.5%). The means of their age and work experience were 31.5 ± 6.71 and 7.31 ± 5.89 years, respectively (Table 1).

**Table 1:** The demographic characteristics of participants

Characteristics		N (%)
Gender	Female	178 (69)
	Male	80 (31)
Marital status	Single	128 (49.5)
	Married	130 (50.5)
Educational level	Bachelor’s	221 (86)
	Master’s and higher	37 (14)
Financial status	Poor	42 (16.5)
	Average	187 (72.5)
	Good	29 (11)
History of affliction by COVID-19	Yes	139 (54)
	No	119 (46)
Employment status	Official permanent	48 (18.5)
	Contractual/Conditional	125 (48.5)
	Agency-affiliated	29 (11)
	Post-graduation service	56 (22)
Age (Years)	Mean ± SD (Range)	31.50 ± 6.71 20–55
Work experience (Years)	Mean ± SD (Range)	7.31 ± 5.89 1–30

The mean scores of MF and SR were 31.73 ± 7.35 and 3.45 ± 0.35, respectively. The highest-scored SR subscale was ethical responsibilities with a mean of 3.67 ± 0.42 and the lowest-scored SR subscale was economic responsibilities with a mean of 2.93 ± 0.62 (Table 2).

**Table 2:** The mean scores of mental fatigue and social responsibility

Variables	Range	Mean	SD	Confidence interval
Mental fatigue	0–44	31.73	7.35	32.83–41.63
Legal responsibilities	1.14–5	3.47	0.56	3.4–3.54
Economic responsibilities	1.43–5	2.93	0.62	2.86–3.01
Ethical responsibilities	2.25–4.89	3.67	0.42	3.62–3.72
Discretionary responsibilities	2.5–5	3.58	0.42	3.53–3.64
Total social responsibility	2.09–4.83	3.45	0.35	3.41–3.5

MF had a significant negative correlation with the legal responsibilities subscale of SR (r = − 0.135) and a significant positive correlation with its economic responsibilities subscale (r = 0.186) (P < 0.05). The correlation of MF with total SR and its ethical and discretionary responsibilities subscales was not significant (P > 0.05) (Table 3).

**Table 3:** The correlation of mental fatigue with social responsibility

Social responsibility	Mental fatigue
Legal responsibilities	r = − 0.135; P* = 0.030
Economic responsibilities	r = 0.186; P* = 0.003
Ethical responsibilities	r = − 0.092; P = 0.139
Discretionary responsibilities	r = 0.013; P = 0.835
Total	r = − 0.002; P = 0.980

*: The results of Pearson’s correlation analysis

The results of the linear regression analysis illustrated that the only significant predictor of SR was good financial status which significantly predicted 4.3% of the variance of SR (P < 0.05). Moreover, no relationship was seen between SR and MF (P > 0.05) (Table 4).

**Table 4:** The results of the linear regression analysis to determine the predictors of social responsibility

Independent variables		B	Beta	t	P value	Confidence interval	${R}^{2}$
Gender	Female	–2.417	–0.089	–1.384	0.168	–5.857, 1.022	0.43
	Male	Reference					
Financial status	Moderate	1.332	0.047	0.593	0.554	–3.094, 5.758	
	Good	6.136	0.154	1.981	0.049	0.035, 12.238	
	Poor	Reference					
Employment status	Contractual/Conditional	2.383	0.095	1.112	0.267	–1.839, 6.604	
	Company affiliated	–2.316	–0.058	–0.770	0.442	–8.241, 3.610	
	Post-graduation service	–0.650	–0.021	–0.264	0.792	–5.500, 4.201	
	Official permanent	Reference					
Mental Fatigue		0.077	0.045	0.699	0.485	_0.141, 0.295	
		Reference					

## Discussion

The study aimed to assess the relationship between MF and SR among nurses who provided care to patients with COVID-19. Findings indicated that the score of MF among these nurses was more than mean score of MF. In the current research, more than half of the nurses who provided care to patients with COVID-19 suffered from MF. This is in agreement with the findings of some previous studies. For example, a cross-sectional study among 2267 nurses on the frontline of COVID-19 care in Wuhan, China, found that 35% of them suffered from fatigue [21]. Another study on 3557 Chinese healthcare workers during the COVID-19 pandemic reported moderate to severe fatigue and showed that the level of fatigue among healthcare workers in the frontline of COVID-19 care was 53% more severe than fatigue among other healthcare workers [38]. Similarly, a study on 442 nurses in Iran showed that COVID-19-related fatigue and anxiety among nurses on the frontline of COVID-19 care were slightly higher than among other nurses, though the difference was insignificant [39]. Care provision in the frontline of epidemic management has always been a risk factor for mental health problems among healthcare workers [40]. Evidence shows that the increased mental workload of nurses during the COVID-19 pandemic negatively affected their performance and behaviors [41]. Moreover, the lack of personal protective equipment, continuous changes in COVID-19 care protocols, and lack of experience in care provision to patients with COVID-19 confronted nurses with great stress [42]. These problems together with fear over affliction by COVID-19 or its transmission to others increased nurses’ MF [43].

Study findings also indicated that the mean score of SR was high. The highest and lowest scored SR subscales were ethical responsibilities and economic responsibilities respectively. In agreement with this finding, a study in Hamadan, Iran, reported an SR mean score of 3.7 ± 0.4 and the lowest and the highest scored SR subscales were economic SR and discretionary responsibilities with a mean of 4 ± 0.4, respectively. That study also showed that 70% of nurses had great SR [18]. Another study on 270 primary healthcare providers in Isfahan, Iran, found that the lowest and the highest scored SR subscales were respectively economic responsibilities and discretionary responsibilities and nurses valued the spiritual aspects of work more than its material aspects [44]. The high mean score of the discretionary responsibilities subscale of SR may be since Iranians are mostly altruistic and their thoughts, behaviors, culture, and lifestyle are greatly influenced by religious beliefs [45, 46]. A study also reported that nurses’ adherence to professional ethics, meaning-seeking, and spiritualties at work can positively affect their SR [47].

We also found that MF had no significant relationship with SR; however, the results showed MF had a significant positive correlation with economic responsibilities. Economic responsibilities are the core of all other aspects of SR [48]. A study showed that the breadwinning role can be associated with mental health problems such as stress and concern over receiving adequate income to afford all family expenses [49]. The positive correlation of MF with economic responsibilities in the present study may be since more than half of the participants were female, while COVID-19-related restrictions caused great role conflict for employed women and were a great shock for them [50]. Moreover, our findings showed that MF had a significant negative correlation with legal responsibilities. A study in Poland revealed that chronic fatigue and stress among healthcare providers could cause them legal problems and hence, improvement of their work conditions was necessary [51]. Nurses are accountable for their care services and hence, legal responsibilities are among the main attributes of professional nursing [52]. A systematic review also showed that not only clinical and organizational factors, but also legal factors can contribute to occupational stress among healthcare providers [53]. Study findings also indicated that MF had no significant correlation with ethical and discretionary responsibilities. Ethical responsibilities refer to adherence to ethical principles, right practice, justice at work, and respect for others’ rights, while discretionary responsibilities refer to being a good citizen [34]. Although workers in all careers should have ethical practice, ethical principles are more important in the nursing profession due to the significant effects of nursing care on patients’ health and recovery. In other words, the nursing profession is based on ethics and nurses need to attempt to provide safe and quality care [54]. Altruism is also a social and ethical value that can improve nursing care quality in any condition [55]. The insignificant correlation of ethical and discretionary responsibilities with MF in the present study may be since these responsibilities are based on internal values and can exert their effects even in difficult conditions. Of course, ethical and discretionary behaviors can also be affected by external factors such as the availability of a good source of financial support [56].

Study limitations.

Among the limitations of this study were the over-crowdedness of hospital wards and the heavy workload of nurses during the COVID-19 epidemic which negatively affected eligible nurses’ tendency towards participation in the study. The fatigue and mental condition of the nurses can affect the results, so it was tried to distribute the questionnaires when the nurses are in a stable state.

## Conclusion

This study shows that nurses who provided care to patients with COVID-19 suffered from MF and MF was not related to their SR, and their level of SR was still high during the COVID-19 pandemic. MF had a significant negative correlation with their legal responsibilities and a significant positive correlation with their economic responsibilities. Healthcare authorities and policymakers need to develop programs for the regular assessment of nurses’ MF and continuous improvement of their mental health. It is suggested that the planners of the health system by designing interventions related to promoting the level of financial and legal independence of nurses provide a basis for satisfaction and reduce the effects of MF. In addition, nurses and health providers can use the results of this study in training sessions with hospital managers and officials, while informing about the importance of SR and its various dimensions, and emphasize their MF. It is recommended to conduct more research on the relationship between the physical, mental and social health of nurses and their SR in challenging situations. More evidence can help design targeted and effective interventions to promote the responsibility of nurses in society.

## Data Availability

The datasets used and analyzed during the present study are available from the corresponding author upon reasonable request.

## List of abbreviations

MF: Mental fatigue
SR: Social responsibility
